# Experimental analysis and numerical fatigue life prediction of 3D-Printed osteosynthesis plates

**DOI:** 10.3389/fbioe.2023.1133869

**Published:** 2023-03-22

**Authors:** Mohsen Nakhaei, Manon Sterba, Jean-Marc Foletti, Laurent Badih, Michel Behr

**Affiliations:** ^1^ Glad Medical SAS, Salon De Provence, France; ^2^ Aix Marseille Université, Université Gustave Eiffel, LBA, Marseille, France; ^3^ Assistance Publique, Hôpitaux de Marseille, Marseille, France

**Keywords:** numerical simulation, fatigue analysis, finite element simulation, biomechanics, additive manufacturing, osteosynthesis plate

## Abstract

The trend towards patient-specific medical orthopedic prostheses has led to an increased use of 3D-printed surgical implants made of Ti6Al4V. However, uncertainties arise due to varying printing parameters, particularly with regards to the fatigue limit. This necessitates time-consuming and costly experimental validation before they can be safely used on patients. To address this issue, this study aimed to employ a stress-life fatigue analysis approach coupled with a finite element (FE) simulation to estimate numerically the fatigue limit and location of failure for 3D-printed surgical osteosynthesis plates and to validate the results experimentally. However, predicting the fatigue life of 3D components is not a new concept and has previously been implemented in the medical device field, though without experimental validation. Then, an experimental fatigue test was conducted using a proposed modification to the staircase method introduced in ISO 12107. Additionally, a FE model was developed to estimate the stress cycles on the plate. The stress *versus* number of cycles to failure curve (S-N) obtained from the minimum mechanical properties of 3D-printed Ti6AI4V alloy according to ASTM F3001-14 to predict the fatigue limit. The comparison between experimental results and fatigue numerical predictions showed very good agreement. It was found that a linear elastic FE model was sufficient to estimate the fatigue limit, while an elastic-plastic model led to an accurate prediction throughout the implant’s cyclic life. The proposed method has great potential for enhancing patient-specific implant designs without the need for time-consuming and costly experimental regulatory testing.

## 1 Introduction

The tendency toward patient-specific medical devices has increased the use of Ti6Al4V 3D-printed surgical implants (Chowdhury et al., 2022; Lam et al., 2022; Tom et al., 2022). Several standards specify requirements for the minimum mechanical properties of Ti6AI4V alloys produced by additive manufacturing (ASTM-F3001-14, ISO 5832-3). These standards do not include minimum requirements in fatigue, although it was previously suggested that the main factor for failure of medical implants is the initiation and propagation of cracks due to cyclic loading (Bowers et al., 2022), referred to as fatigue damage. In addition, non-homogeneous porosity and rough surfaces due to partial melting of some particles were also more recently reported as affecting fatigue life (i.e., the number of cycles or repetitions *N* of a given stress level that a design can endure before it fails) by creating notches with stress concentration, which increases the risk of fatigue damage (Bordin et al., 2017; Almansoori et al., 2020; Zhu et al., 2023). Over the past decades, the additively manufactured Ti-6Al-4V alloy has been extensively studied due to its popularity in the manufacturing industry. Researchers have focused on the influence of process parameters on the as-built and heat-treated microstructures (He et al., 2019), as well as on mechanical properties like ultimate tensile strength, tensile yield strength, and elongation (Liu et al., 2019; Tao et al., 2019), fatigue properties (Benedetti et al., 2018), and residual stresses (Bartlett and Li, 2019). However, fatigue failures in additively manufactured implants remain a concern, and there are insufficient long-term clinical studies on the biomechanical performance of such implants (Jehn et al., 2020). Besides the need for clinical data, pre-clinical studies must also be performed to evaluate the efficiency and safety of the implants compared to standard implants used for similar clinical conditions.

Resistance to fatigue failure is usually evaluated by cyclic loading tests on a reduced number of samples, as described, for example, in the ISO-14801 standard for dental implants and in the ISO 7206-4 standard for hip joint prostheses. These standards have contribute to reduce significantly the failure rate of implants (Griza et al., 2008) but the evaluation under these standards remains time consuming and expensive. Therefore, the development of a validated fatigue simulation tool would result in time and cost savings, and would be better adapted to patient-specific and 3D-printed medical devices that are usually produced in one or very few samples.

Previous attempts to predict fatigue failure in medical implants using numerical models can be found in literature. In a recent study (Ortega et al., 2020), a finite element analysis (FEA) was performed that showed a stress concentration consistent with the experimentally observed location of crack initiation. In addition, the fatigue life of a hip implant and orthopedic plates were also recently investigated by comparing experiments and a linear elastic FEA in (Babić et al., 2020), (Parr et al., 2021). In these studies, FEA was able to accurately predict the location of fatigue crack initiations that were observed in the experiment. A multidimensional and comprehensive analysis of the fatigue failure mechanism of a selective laser melting (SLM) fabricated Ti6Al4V orthopedic mandibular plate using FEA was also previously proposed (Shi et al., 2021). The FEA was able to predict the location of peak stress, although the value was far below the fatigue strength of the material, i.e., indicating a low risk of failure.

All these studies succeeded in predicting weak regions of complex 3D-printed titanium structures (e.g., implants or stabilization plates) based on stress and/or strain thresholds and gradients in FEA, always in one single static loading simulation. This type of approach allows to identify the region where and how the structure fails in a qualitative manner, but were not able to predict fatigue limit, i.e., the fatigue life of an implant at a certain level of peak loading force.

In parallel, it is widely admitted that the number of loading cycles is a determining factor for failure (Stephens and Fuchs, 2001; Zahavi and Torbilo, 2019). In this context, the so-called stress-life approach provides an analytical alternative to estimate the fatigue life of a sample under cyclic loading (Dowling, 2004). This method has been developed and widely used in the aerospace field (Dowling, 2004; Williams and Fatemi, 2007). This stress-life approach has been also recently used to predict fatigue life of medical implants (Ziaie and Khalili, 2021) but to the best of our knowledge without validating the results experimentally.

Using the strengths of these previous studies, the aim of this study is to predict by FEA the location of peak stress, in order to identify the fatigue failure location in 3D-printed osteosynthesis plate, and also the fatigue life. To do so, an experimental fatigue test was developed, based on a modified staircase method to estimate the fatigue limit, i.e., the force level below which an infinite number of loading cycles can be applied to a material without causing fatigue failure, of a specific model of SLM-fabricated osteosynthesis plate. In parallel, the experimental test conditions were numerically reproduced and used to predict fatigue failure location by FEM and calculate fatigue life. Once validated, the method should provide a simulation tool to predict the fatigue limit force (i.e., the maximum force the implant can withstand for a given number of loading cycles) without performing expensive normative tests.

## 2 Materials and methods

**FIGURE 1:**
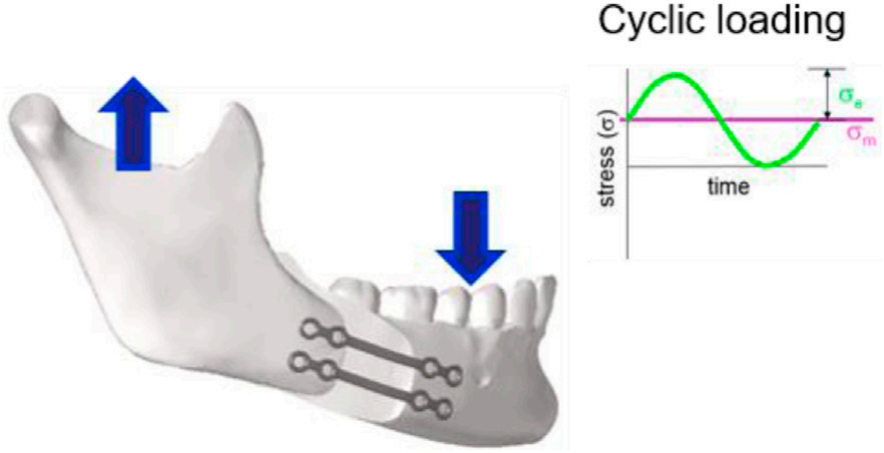
In-service loading condition of the osteosyntheses plates used in pairs. The arrows in the image illustrate the main components of the occlusal force. Cyclic loading is characterized by stress load cycles with a constant amplitude ( $\sigma_{a}$ ) and mean stress ( $\sigma_{m}$ ).

This study was performed with a special osteosynthesis plate model that is considered the standard plate for fixation of bilateral sagittal split osteotomy. The plate, shown in Figure 1, is offered by several manufacturers.

Our approach consists of two consecutive steps. First, the fatigue limit of the plate was estimated by performing cyclic bending tests. Secondly, a numerical model of the plate was developed, and further coupled to a fatigue limit estimation method. Results were finally compared to those of the experimental bending tests.

### 2.1 Cyclic bending tests

#### 2.1.1 Osteosynthesis plate characteristics

The generic osteosynthesis plate was fabricated by Selective Laser Melting (SLM) from a titanium alloy (Ti6Al4V ELI) using an SLM® 125 HL - SLM Solutions Machine. To ensure the best quality of Ti6Al4V Grade 23 ELI in accordance with the ASTM F3001-14 standard, the suggested parameters of SLM Solutions were used. The particle size had a range of 20-63 µm with a spherical shape, and the build-up rate was 18.14 cm³/h with a laser power of 400W. Subsequently, the plate was subjected to heat treatment in accordance with AMS-H-81200 which involved annealing in vacuum at 800 °C ± 14 °C for 2 h, followed by cooling in air, and then blasting.

This ensured that the manufacture plate met the specifications of ASTM F3001-14 and ISO 5832-3 standards, which required a minimum tensile strength of 860 MPa, a yield strength at 0.2% offset of not less than 795 MPa, and a maximum elongation before fracture of at least 10%. Additionally, the elastic modulus for Ti6Al4V ELI is 110 GPa.

#### 2.1.2 Conditions of use

The conditions of use were defined according to *a priori* knowledge on what could be considered a “normal use”, as detailed here after. Osteosynthesis plates used in the mandibular and maxillary regions are mainly loaded by forces associated with occlusion and jaw opening (Ellis and Throckmorton, 2005). Figure 1 shows an example of the plate fixation in which the transverse bending load is the main loading condition.

In healthy individuals, the number of masticatory cycles was estimated at 500,000 to 1 million per year (Buschang et al., 2000; Wintergerst et al., 2006; Prados-Privado et al., 2019), i.e., approximately 10,000 to 20,000 per week. During these cycles, the functional physiological forces remain lower than the maximum reported bite forces (maximum occlusion) (Peck, 2016), and depend on various factors such as the consistency of the bowl (Shimada et al., 2012).

After orthognathic or post-traumatic surgery with osteosynthesis plates, a first period of 4–8 weeks is required for clinical stabilization (also called primary stabilization) (Reitzik and Leyden, 1983; Gerlach and Schwarz, 2002), followed by a secondary stabilization phase, where the anatomical and mechanical properties of mature bone are fully restored (Reitzik and Leyden, 1983; Ueki et al., 2014). The duration of secondary stabilization phase highly depends on the patient (Reitzik and Leyden, 1983; Ueki et al., 2014). The newly formed bone tissue takes over the loads, and as a result progressively reduces the loads carried by the plate (Throckmorton et al., 1996). For all these reasons, a total number of N_max_ = 100,000 loading cycles was considered in this study as the number of cycles the plate should sustain without fatigue failure.

#### 2.1.3 Experimental setup

**FIGURE 2:**
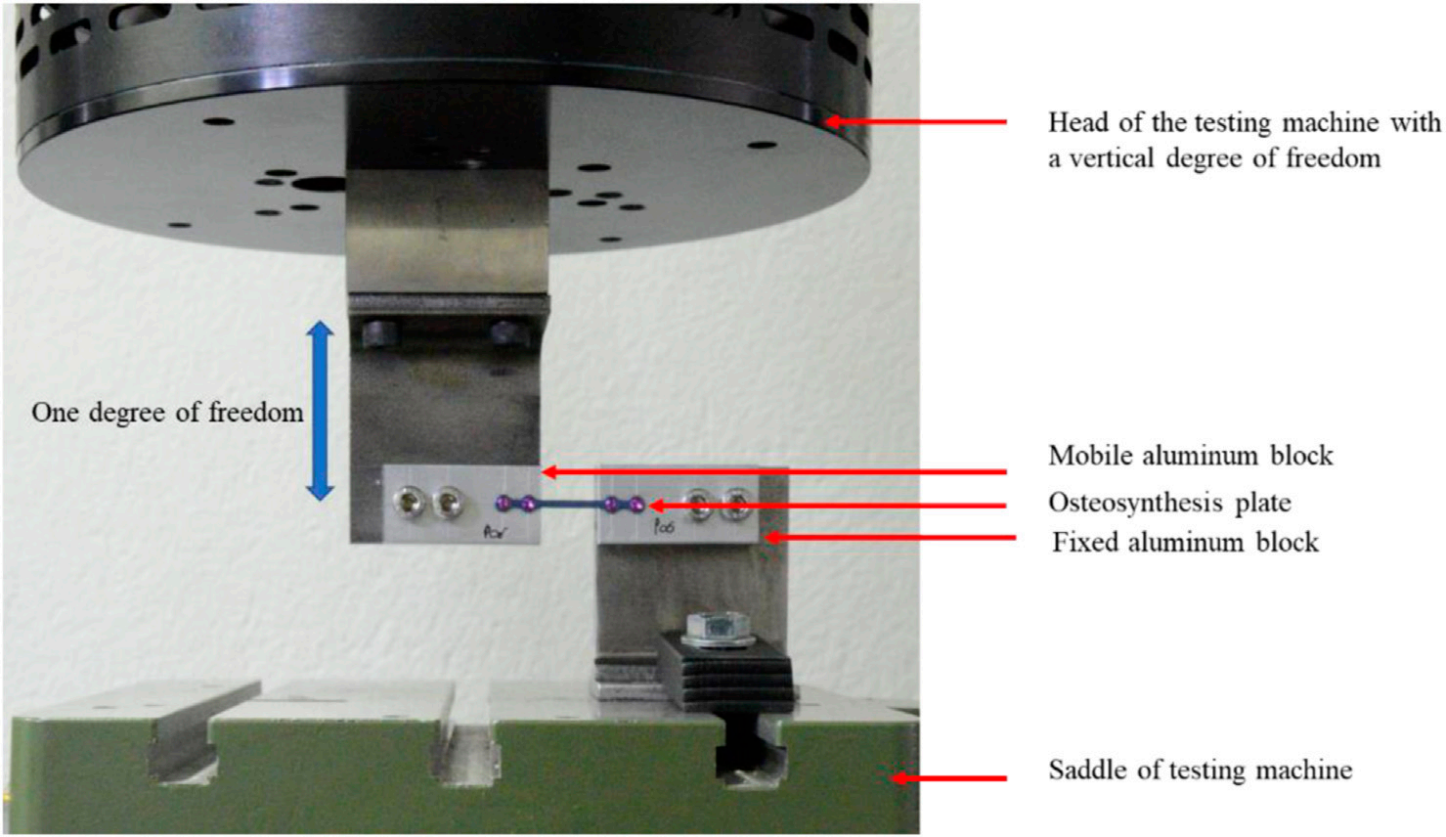
Cyclic bending experimental setup of the osteosynthesis plate. The plate was fixed to two aluminum blocks with four self-tapping titanium miniscrews (2 mm). One aluminum block was fixed to the saddle of the testing machine, while the other one was attached to the head of the machine and had a vertical degree of freedom.

As there was to our knowledge no standard available to evaluate the fatigue performance of this type of osteosynthesis plates (less than 50 mm length), we attempted to replicate the critical in-service condition of use of the plate so that the simplified test setup implies the main loading on the implant. The plate was screwed into two aluminum blocks using four 2 mm diameter screws (Self-Tapping Ti6Al4V Miniscrews) in 1.7 mm diameter pilot holes. One block was fully constrained while the other was mounted on the axis of an Acumen 3 (MTS System Corp, Chicago, United States), therefore limited to one degree of freedom in vertical translation (see Figure 2).

#### 2.1.4 Loading conditions

**FIGURE 3:**
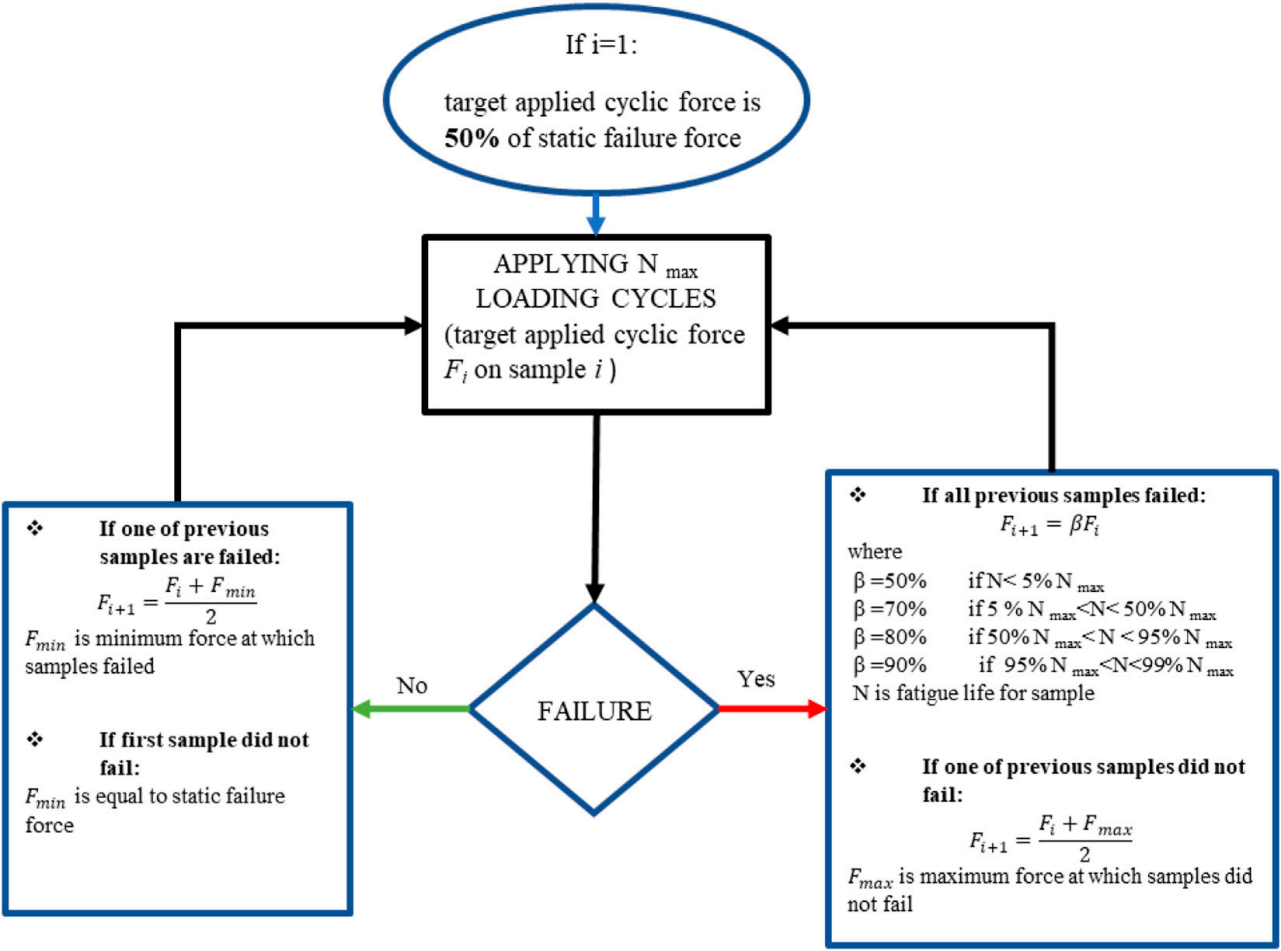
Modified staircase method derived from the ISO 12107 standard. Testing begins at 50% of the static force at failure (127.3 N), and the next target applied cyclic force is calculated based on the obtained fatigue life.

In order to estimate the fatigue life of the generic plate, the general method detailed in the ISO 12107 and ISO 14801 standards consists in performing cyclic loadings on a set of samples at various target applied cyclic force (*F*_*i*_). The first sample is tested at 80% of the static failure force, i.e., a value that is expected to lead to an early failure of the sample (80% of the static failure force for this plate is 203.6 N). This force is then gradually decreased according to a so-called staircase method, as detailed in the ISO 12107 standard, until three specimens survive the target number of cycles (here *N*_*max*_ = 100,000 cycles). However, in the method used in this paper, shown in Figure 3, the target applied cyclic force (*F*_*i*_) was defined for each iteration *i* of the experimental test. It was derived from the staircase method mentioned here above and modified to optimize the number of samples requested.

In the proposed method, the value of the target applied cyclic force for iteration *i+1* (*F*_*i+1*_) is calculated from the value at iteration *i* (*F*_*i*_) and the previous number of cycles before failure, rather than using a fixed percentage of *F*_*i*_ as defined in the ISO standard. Furthermore, the initial fatigue test is conducted at 50% of the static force at failure (127.3 N), instead of 80% as prescribed by the ISO standard (203.6 N).

For each iteration, the testing machine was driven with imposed force conditions. The test began with a load ramp at a rate of 1 N/min, starting from 0 and progressing to a preconditioning force value of 15 N. The preconditioning force was calculated based on 10% of the maximum dynamic load applied to the plate (127.3 N). This force was further adjusted based on the capacity of the testing machine’s load cell. This preconditioning was followed by a sinusoidal cyclic loading, which ranged from 15 N to the target applied force *F*_*i*_, at a frequency of 13 Hz. This frequency was determined to optimize the test duration while minimizing the dynamic effect on force measurements, which was determined through preliminary tests. The test was terminated at either plate failure or when *N*_*max*_ = 100,000 was achieved. The tests were conducted at room temperature.

In this method the number of samples was considered as sufficient if the regression model would fit to the experimental results with a *p*-value lower than 0.05.

#### 2.1.5 Numerical analysis

**FIGURE 4:**
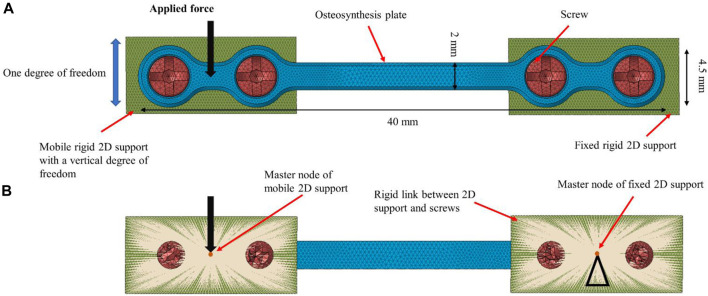
Finite element model of osteosynthesis plate (40 × 4.5 × 1 mm). **(A)**: Front view and **(B)**: back view. The screws were fixed to the 2D supports using rigid links. A force was applied to the master node of the left 2D support, while the right 2D support was fully constrained. The contact between the plate and screws was modeled as sliding.

A finite element model of the generic osteosynthesis plate was developed with the 3D tetrahedral elements in first order with 4 nodes and one integral point at the center of the element. The same element type was used to discretize the screws geometry. For the fixed rigid 2D support plates, 2D triangle elements were used with three integration points at the standard Gauss point locations. Boundary conditions were defined according to the experiments, as illustrated in Figure 4:

The right side of the plate was fixed to a rigid 2D support by means of a sliding contact (friction coefficient set to 0.3 (Cordey et al., 2000; Raja Izaham et al., 2012)) between the screw and the osteosynthesis plate as well as between the plate and the 2D support, while a rigid link was defined between the screws and the 2D supports. The 2D support itself was constrained in all degrees of freedom.

The left side of the plate was similarly attached to a rigid 2D support that was constrained in all degrees of freedom except vertical translation.

**FIGURE 5:**
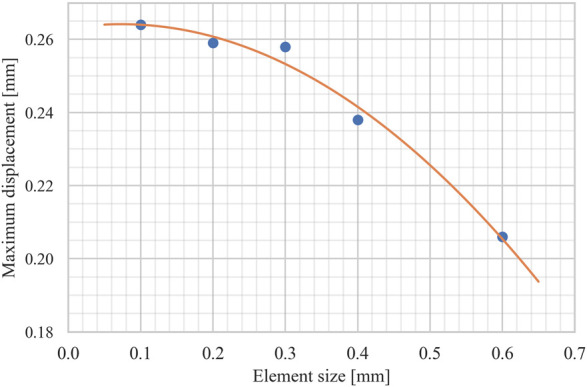
The mesh convergence analysis results. The maximum displacement of the plate was measured for different element sizes, ranging from 0.6 mm to 0.1 mm.

The SLM-manufactured Ti6Al4V plate was assumed elastic and isotropic with elastic modulus of 110 GPa. A non-linear implicit FE solver (Optistruct v2021, Altair, Troy, United States) was used to account for the geometric and contact non-linearity under the applied static loads. Prior to further analysis, the mesh convergence analysis was performed by decreasing the element size and analyzing the impact of this process on the maximum displacement of the plate (see Figure 5). Then, a 0.3 mm mesh size was considered to discretize the model in order to obtain the optimal balance between accuracy and computational costs. Table 1 shows the material properties and detailed number of elements for each component.

**TABLE 1:** Element type and mechanical properties (Elastic modulus and Poisson’s ratio) of different components.

Component	Element type	Number of elements	Mechanical properties
Osteosynthesis Plate	3D tetrahedral	37949	E = 110 GPa, and ν = 0.34
Screw	3D tetrahedral	1854	E = 110 GPa, and ν = 0.34
2D support	2D triangle	3512	Rigid body

In order to estimate the fatigue life from the model, static loading simulation was performed in the condition of previously performed experimental test detailed in Figure 3, i.e., the stress state on the plate evaluated for each tested target applied cyclic force (*F*_*i*_). Each stress result was then used to estimate fatigue life, as described in the following as the stress-life approach.

#### 2.1.6 Static loading simulation

For each experimental iteration *i*, a static load equal to *F*_*i*_ was imposed to the master node of the left support plate rigid body in the vertical downward direction (see Figure 4). Then, the resulting peak value and position of the maximum absolute principal stress (APS) were recorded for each corresponding experimental iteration *i*. The APS criterion was selected as it is the maximum normal stress that the element will ever experience under the specified applied loads after any transformation. The location of the peak APS value in the numerical simulation can inform us on the plate locations where crack initiation and propagation are likely. In addition, for each experimental iteration the maximum APS value at preconditioning force (i.e. 15 N) considered as $\sigma_{\min}$ and the maximum APS value at target applied cyclic force (*F*_*i*_) considered as $\sigma_{\max}$ . These stress values were used to calculate the stress amplitude $\sigma_{a}$ , assuming a constant stress amplitude, and mean stress $\sigma_{m}$ on the plate as detailed in Eqs 1 and 2.

$$\begin{array}{c} \sigma_{a}=\frac{\sigma_{\max}-\sigma_{\min}}{2} \end{array}$$ (1)

$$\begin{array}{c} \sigma_{m}=\frac{\sigma_{\max}+\sigma_{\min}}{2}. \end{array}$$ (2)

Therefore, these two stresses extremum $\sigma_{\min}$ and $\sigma_{\max}$ calculated from FE analysis allow to obtain the undergo stress cycle on the plate.

#### 2.1.7 Stress-life approach

Based on the stress-life approach, the fatigue life under different stress cycles is characterized by the stress amplitude and mean stress. Furthermore, stress-life approach is originally developed for the stress cycles with zero mean stress ( $\sigma_{m}=0$ ) (Sendeckyj, 2001), however the plate is under stress cycles with non-zero mean stress. Therefore, a correction method is necessary to consider the mean stress effects on the fatigue life prediction. The first correction method used in this study was the one detailed by Goodman (Stephens and Fuchs, 2001), known as the Goodman mean stress correction, in the form given in Equation 3.

$$\begin{array}{c} \frac{\sigma_{a}}{\sigma_{w}}+\frac{\sigma_{m}}{\sigma_{u}}=1 \end{array}$$ (3)

where $\sigma_{w}$ is the fatigue limit for completely reversed stress cycle (when, $\sigma_{m}=0$ ) and $\sigma_{u}$ is the ultimate tensile stress. The Goodman mean stress correction usually leads to an under-estimated fatigue life (Sendeckyj, 2001; Asada et al., 2016; Liu et al., 2020). Therefore, a second correction method was also used which is the one introduced by Gerber (Stephens and Fuchs, 2001), given in Eq. 4.

$$\begin{array}{c} \frac{\sigma_{a}}{\sigma_{w}}+{\left(\frac{\sigma_{m}}{\sigma_{u}}\right)}^{2}=1 \end{array}$$ (4)

In the stress-life approach, it is common to use stress *versus* number of cycles to failure (S-N) curves and statistical models such as Basquin, Strohmeyer or Bastenaire to fit to the experimental data. Among these statistical models, the Basquin model as described in (Zahavi and Torbilo, 2019) was chosen here, as only two parameters $\sigma_{f}^{\prime }$ and *b* are sufficient to fit the S-N curve (Eq. 5),

$$\begin{array}{c} \sigma_{a}=\sigma_{f}^{\prime }{\left(2N\right)}^{b} \end{array}$$ (5)

where $\sigma_{f}^{\prime }$ is the fatigue strength coefficient and *b* is the fatigue strength exponent which are cyclic material parameters. To obtain these parameters, the S-N curve for Ti6AL4V ELI alloy was derived from standard properties of titanium alloy, estimated from the ultimate tensile stress $\sigma_{u}$ and the elastic modulus of the material, according to previous studies (Lee et al., 2005; nCode Theory Guide, n. d.) as detailed here after.

To ensure that no significant plastic behavior occurs, the life axis (*N*) of S-N curve was confined to life greater than 1,000 cycles. The stress at *N* = 1,000 cycles *S*_*1*_ is given by Eq. (6),

$$\begin{array}{c} S_{1}=0.8\times \sigma_{u}, \end{array}$$ (6)

which is a stress value close to material’s yield stress. Moreover, *N* = 10^6^ is considered as the endurance limit number of cycles (*Nc1*) for titanium alloy such that if the sample survives *Nc1* cycles, an infinite number of loading cycles can be applied to the material without causing fatigue failure. The stress value at Nc1 is given by Eq. 7:

$$\begin{array}{c} S_{2}=0.307\times C_{f}\times \sigma_{u} \end{array}$$ (7)

In order to take into account the S-N curve for Ti6AL4V ELI alloy with specific manufacturing method (3D-pringing), and surface treatment processes, a factor *C*_*f*_, ranging from 0 to 1, was introduced to correct the stress value at *Nc1*. The factor *C*_*f*_ took into consideration the most influential factors on fatigue life prediction such as surface roughness (McKelvey et al., 2012; Greitemeier et al., 2016), stress concentration (Ciavarella and Demelio, 1999), and material defects (Yadollahi et al., 2018).

Then, given *S*_*1*_, *S*_*2*_, and *Nc1* one can compute the parameters of Basquin model (*b* and $\sigma_{f}^{\prime }$ ) with Eqs 8 and 9,

$$\begin{array}{c} b=\frac{\log\left(S_{2}\right)-\log\left(S_{1}\right)}{\log\left(Nc1\right)-3}\; \end{array}$$ (8)

$$\begin{array}{c} \sigma_{f}^{\prime }=\frac{S_{1}}{{\left(Nc1\right)}^{b}} \end{array}$$ (9)

Finally, the fatigue strength coefficient $\sigma_{f}^{\prime }$ and the fatigue strength exponent *b*, through Basquin model (Eq. 5) make it possible to relate the applied stress amplitude on the plates ( $\sigma_{a}$ ) and fatigue life *(N*).

## 3 Results

### 3.1 Experimental results

Prior to fatigue tests, six plates were tested on the same experimental setup. The mobile side of the plate was moved downward under displacement control until failure occurred. The static failure forces obtained from the six samples were 210.5 N, 299.6 N, 262.0 N, 236.6 N, and 263.9 N. The average static failure force was calculated to be 254.5 N, with a standard deviation of 29.8.

Then, six plates were tested under dynamic loading based on the loading conditions detailed above, and using the modified staircase method (see Figures 2 and 3). Table 2 shows the target applied cyclic force, fatigue life, and the location of failure for the six tested plates.

**TABLE 2:** Experimental number of cycles to failure (*N*) for the six samples tested at different force levels up to 100,000 cycles.

Sample identification	Applied target cyclic force (F_a_)	Fatigue life (N)	Observation
E01	127.3	1,230	failure at bridge mobile side
E02	63.6	23,262	failure at bridge mobile side
E03	44.5	78,567	failure at bridge mobile side
E04	35.6	100,000	No failure
E05	40.1	100,000	No failure
E06	42.3	100,000	No failure

**FIGURE 6:**
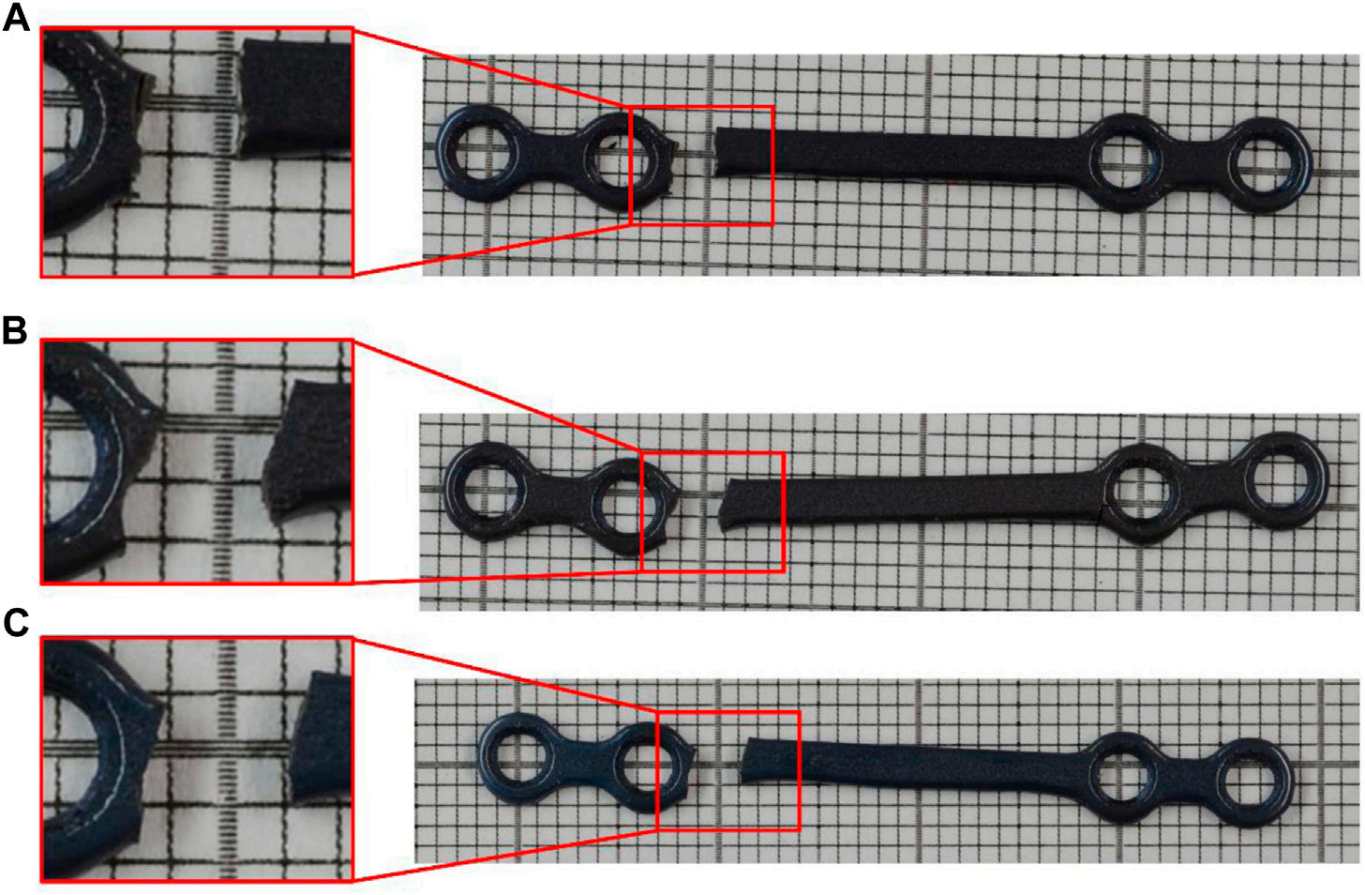
Failure profile of the osteosynthesis plates. **(A)**: sample E01 with 127.3 N applied cyclic Force, **(B)**: sample E02 with 63.6 N applied cyclic Force, and **(C)**: sample E03 with 44.5 N applied cyclic Force.

The last three samples did not fail before reaching the *N*_*max*_ target of 100,000 cycles. The failure profile was similar for the three first samples and is illustrated in Figure 6. All failures happened at the same location on the moving side of the plate, on the bridge.

### 3.2 Numerical results

The experimental target applied forces (*F*_*i*_) were then applied on the FE model to compute the maximum stress magnitude and location for each sample. Table 3 gives the peak APS values and location for the six tested plates at the applied and preconditioning force.

**TABLE 3:** Peak APS at applied and preconditioning load, and peak stress location in the finite element model.

Applied target cyclic force (F_a_)	Peak APS (MPa) at F_a_	Max. APS (MPa) at 15N preconditioning	Max. APS location
127.3	1824.5	220.1	Bridge mobile side
63.6	940	220.1	Bridge mobile side
44.5	658.6	220.1	Bridge mobile side
35.6	526.2	220.1	Bridge mobile side
40.1	593.2	220.1	Bridge mobile side
42.3	525.9	220.1	Bridge mobile side

**FIGURE 7:**
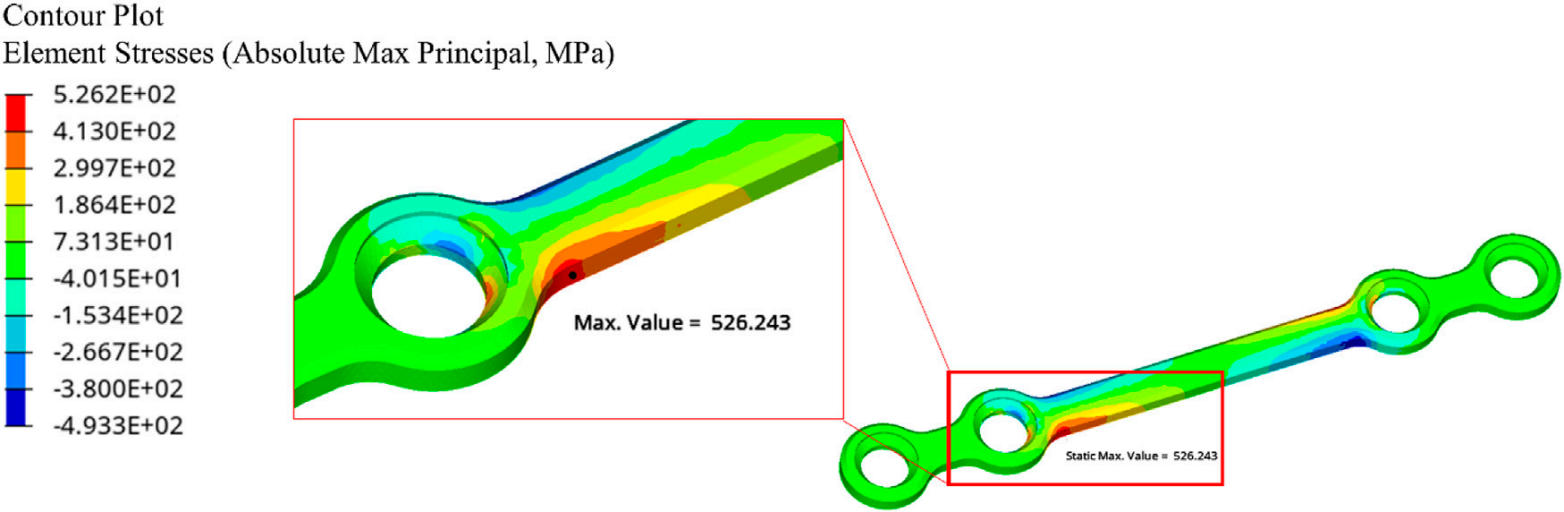
Absolute principal stress distribution and maximum location for a 35.6 N applied force.

The finite element results showed a peak APS value at the plate’s bridge on the mobile side (see Figure 7).

Comparing Figure 6 and Figure 7 shows the peak APS value in the FE model occurs in the same region as the failure in the experimental tests.

Then peak APS value corresponding to the applied and preconditioning force were used to compute the amplitude and mean stress according to Eqs 1 and 2. The Goodman and Gerber mean stress corrections were calculated respectively from Eqs 3 and 4, considering *C*_*f*_ = 1. Finally, the fatigue life N was computed from Basquin model (Eq. 5). The numerically estimated fatigue life using Goodman and Gerber corrections for each sample were compared with experimental results in Table 4. Note that the fatigue life was limited to 100,000 cycles (as in the experiments) and higher obtained fatigue life estimations were truncated to this value.

**TABLE 4:** Numerical and experimental fatigue life for the six samples.

Sample identification	Fatigue life (N) from goodman method	Fatigue life (N) from gerber method	Fatigue life (N) from experiment
E01	5	5	1,230
E02	313	3893	23262
E03	24329	100,000	78567
E04	100,000	100,000	100,000
E05	74066	100,000	100,000
E06	42127	100,000	100,000

**FIGURE 8:**
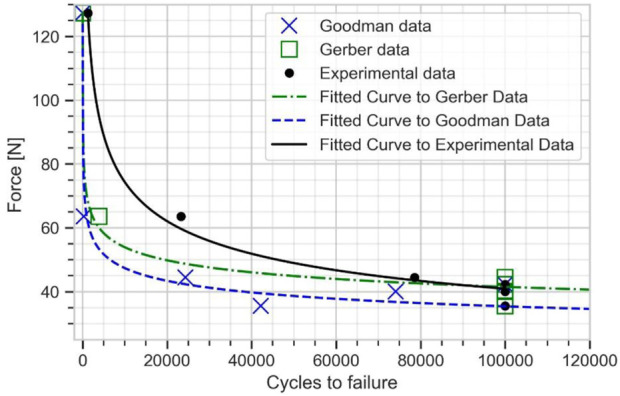
Force vs. number of cycles to failure curve for the plate from experimental data, Goodman, and Gerber mean stress correction using elastic FE model.

The numerical estimation of cycles and the experimental data were fitted to Basquin model to plot the force *versus* number of cycles to failure curve (see Figure 8). The experimental fatigue limit estimation was 40.92 N at 100,000 cycles. The fatigue limit estimated from Goodman mean stress correction was 35.43 N, while a value of 41.52 N was found with Gerber mean stress correction. The Gerber mean stress correction leads to a fatigue limit in good agreement with the experimental results with approximately 2% error.

## 4 Discussion

The results of the fatigue experimental tests showed that the 3D-printed osteosynthesis plates made of Ti6Al4V alloy has a fatigue limit of 40.92 N, considering *N*_*max*_ = 100,000, which was within the 5% interval of the obtained failure force for the plate at 100,000 cycles. This confirms the reliability of the modified staircase method for assessing the fatigue life of 3D-printed components. Furthermore, it was established that the number of test samples required for the modified staircase method to obtain the fatigue limit can be as low as six, compared to the twelve samples required by the classical staircase method in ISO 12107. This implies that the modified staircase method is more efficient and economical than its classical method, making it a preferable option for fatigue testing of 3D-printed components.

The average static failure force of the plate obtained as 254.5 N, which was higher than the maximum occlusion force values of orthognathic surgery patients at 6 weeks post-surgery reported as 105 ± 75 N (Tate et al., 1994; Harada et al., 2000). Therefore, there is no risk of failure due to sudden peak occlusion force, as these plates are used in pair on the patient. Furthermore, to determine the maximum dynamic force the plate can withstand up to 100,000 loading cycles, the experimental test was conducted using a modified version of the staircase method in which dynamic force was applied at different values. The fatigue limit of the plate was determined to be 40.9 N after 100,000 loading cycles, which is sufficient to meet clinical needs. This is because the plate is primarily used to provide initial stabilization to the bone while newly formed bone tissue takes over the load, thus gradually reducing the forces applied to the plate. Fluid intake regimes during the healing process also help to reduce the load placed on the plate.

The numerical evaluation of fatigue limit at 100,000 cycles lead to a fatigue limit of 41.52 N for the plate. The numerical prediction of the fatigue life for the plates was found to be accurate, with an error of only around 2%. The peak APS value at the fatigue limit force was 613 MPa, which is lower than the yield strength of the plate material Ti6AL4V alloy (795 MPa) (F42 Committee, n.d.). Therefore, the plate operated under purely elastic strain in the high cycle fatigue domain, confirming the use of stress-life approach method and its accuracy in its validity domain. Additionally, this serves as further evidence that material yield strength are not a reliable indicator of fatigue failure, as also experimentally observed previously (Shi et al., 2021).

For specimens E01 and E02, which were respectively tested under a cyclic load of 127.3 and 63 N, the peak APS value exceeded the yield strength of 795 MPa, indicating that the plates were partially undergoing plastic strain. Thus, these two samples were outside the domain of validity of the stress-life approach (Stephens and Fuchs, 2001), and the method was not able to accurately predict the fatigue life. This was evidenced by the dispersion between experimental and numerical results at high load levels (see Figure 8).

### 4.1 Constitutive analysis of cyclic deformation behaviors

The validity domain of the stress-life or strain-life approaches can be investigated using the transition fatigue life (*N*_*t*_) (i.e., the number of loading cycles where the elastic and plastic strain are equal) (Nguyen et al., 2022). If the number of loading cycles is lower than *N*_*t*_, then the deformation is dominated by plastic strain and should be addressed by a strain-based approach. On the other hand, if the number of cycles is higher than *N*_*t*_, then the deformation should be addressed by a stress-based approach (Nguyen et al., 2022). The total strain amplitude ( ∆ϵ/2 ) is the sum of elastic and plastic strains amplitude which is described in detail in (Manson, 1979). Basically, the life relation is given in the form:

$$\begin{array}{c} \frac{∆\epsilon }{2}=\frac{{∆\epsilon }_{e}}{2}+\frac{{∆\epsilon }_{p}}{2} \end{array}$$ (10)

where elastic strain amplitude in equation (10) is given by the strain form of the Basquin’s equation as (Zahavi and Torbilo, 2019):

$$\begin{array}{c} \frac{{∆\epsilon }_{e}}{2}=\frac{\sigma_{f}^{\prime }}{E}{\left(2N\right)}^{b} \end{array}$$ (11)

where $\sigma_{f}^{\prime }$ is fatigue strength coefficient and *b* is fatigue strength exponents. $\epsilon_{e}$ is elastic strain, and *E* elastic modulus of the material. The plastic strain amplitude in equation (10) is given by Coffin-Manson’s equation as previously proposed in literature (Manson and Hirschberg, 1970; Coffin, 1978):

$$\begin{array}{c} \frac{{∆\epsilon }_{p}}{2}=\epsilon_{f}^{\prime }{\left(2N\right)}^{c} \end{array}$$ (12)

where $\epsilon_{f}^{\prime }$ is fatigue ductility coefficient, *c* is fatigue ductility exponents, and $\epsilon_{p}$ is plastic strain. The intersection of the elastic strain curve (Basquin’s equation) and plastic strain curve (Coffin-Manson’s equation) clearly demonstrates the transition life cycle ( $N_{t}$ ) from a plastic-dominated fatigue regime to an elastic-dominated fatigue regime (Nguyen et al., 2022), by substituting $\epsilon_{e}=\epsilon_{p}$ as follows,

$$\begin{array}{c} {2N}_{t}={\left(\frac{\epsilon_{f}^{\prime }E}{\sigma_{f}^{\prime }}\right)}^{\frac{1}{b-c}} \end{array}$$ (13)

**FIGURE 9:**
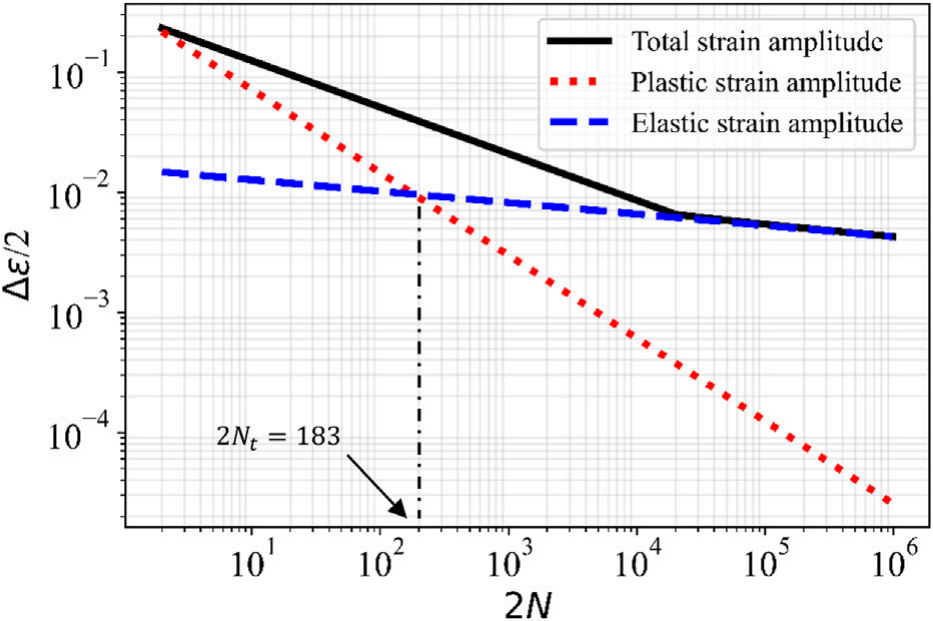
Total, plastic, and elastic strains amplitude *versus* number of reversed cycles to failure (when, σ_m_=0).

This transition life cycle is typically considered as the transition between low and high cycle fatigue for the material (Nguyen et al., 2022). In the high cycle fatigue regime above the *N*_*t*_ values, the fatigue strength ( $\sigma_{f}^{\prime }/E$ ) is the dominant factor in the fatigue performance of materials. In the low cycle fatigue regime, in which the plastic strain is the dominantfactor, the fatigue ductility ( $\epsilon_{f}^{\prime }$ ) dictates the fatigue performance of the material (Nguyen et al., 2022; Kim et al., 2023). The Equation (10) was plotted for Ti6Al4V material in Figure 9 in black solid line (total strain amplitude), this figure also indicates the elastic strain amplitude (Basquin’s equation) and plastic strain amplitude (Coffin-Manson’s equation) during fatigue life.

The standard strain-number of cycle (E-N) properties of the titanium alloy obtained from previous studies (Lee et al., 2005; nCode Theory Guide, n. d.). These studies suggest the fatigue ductility coefficient and exponent as $\epsilon_{f}^{\prime }=0.35$ and c=−0.69 . In addition, the fatigue strength exponent suggested as b=−0.095 and the fatigue strength coefficient can be computed as:

$$\begin{array}{c} \sigma_{f}^{\prime }=1.67\times \sigma_{u} \end{array}$$ (14)

Therefore, the transition life cycle for a Ti6Al4V ELI with the elastic modulus (E) of 110 GPa and a tensile strength ( $\sigma_{u}$ ) of 860 MPa can be obtained as ${2N}_{t}=183$ cycles. This value is comparable with the transition life cycle reported for a Ti6Al4V parts manufactured by SLM as ${2N}_{t}=164$ cycles by Borrego et al. (Borrego et al., 2018). Thus, at 100,000 load cycles that the plate should sustain without fatigue failure the elastic deformation denominates and the strass life approach is valid. It is important to note that in this study the S-N and E-N curves are truncated to 10^6^ life cycles, as we considered an infinite fatigue life for the titanium alloy after this numbery of life cycle (Lee et al., 2005).

### 4.2 Fatigue life assessment considering elastic-plastic deformation

In former fatigue life assessment presented in Section 3.2, stress amplitude on the plate consists of only elastic stress ( $\sigma_{a}=\sigma_{a}^{e}=E\epsilon_{e}$ ). However, the total stress amplitude is the additive decomposition of two stress tensors components, read as follows (Weissman and Sackman, 2011),

$$\begin{array}{c} \sigma_{a}=\sigma_{a}^{e}+\sigma_{a}^{p} \end{array}$$ (15)

The first component, $\sigma_{a}^{e}$ , depends only on the elastic strain and, consequently, is the elastic stress amplitude. The second term, $\sigma_{a}^{p}$ , depends on the describing hardening kinematic, which, therefore, represent plastic stress. It is important to note that $\sigma_{a}=\sigma_{a}^{p}$ implies that $\epsilon =\epsilon_{p}$ (Weissman and Sackman, 2011). Therefore, it is possible to consider both elastic and plastic deformation into Basquin’s model by considering the new stress amplitude into Equation (5).

To account for the partial plastic deformation of the plate at high levels of applied forces, an elasto-plastic FE model was developed, considering a linear hardening behavior of the material from its yield stress (765 MPa) up to its ultimate strength (860 MPa), in accordance with ASTM F3001-14 standard which define 10% elongation as the limit. Then, the peak APS value from the modified FE model was used in the stress-life approach to estimate the fatigue life. The results are shown in Table 5.

**TABLE 5:** Maximum APS from elasto-plastc finite element model and computed fatigue life.

Sample identification	Max. APS (MPa) at F_a_	Fatigue life (N) from goodman method	Fatigue life (N) from gerber method
E01	1,007.4	115	1738
E02	854.9	1,117	10768
E03	658.6	24329	100,000
E04	526.2	100,000	100,000
E05	593.2	74066	100,000
E06	525.9	42127	100,000

The results from the elasto-plastic FE model showed a significant reduction in the peak APS value for both specimens. This reduction was due to the plastic deformation of the plate, which allowed for a redistribution of the stress along the material and thereby mitigating the peak stress. The elasto-plastic FE model was then used to predict the fatigue life.

**FIGURE 10:**
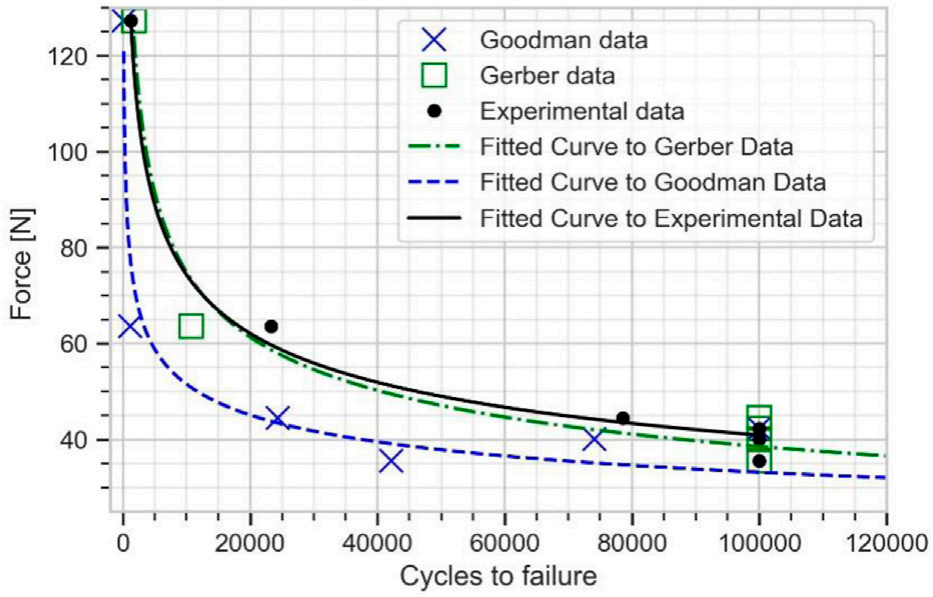
Force vs. number of cycles to failure curve for the plate from experimental data, Goodman, and Gerber mean stress correction using elasto-plastic FE model.

The numerical fatigue life estimated from this elastic-plastic FE model was fitted into Basquin model to plot the plate fatigue life curve for the plate (see Figure 10). This fatigue life curve is used to determine the fatigue limit of the plate. The fatigue limit estimated from Goodman mean stress correction was obtained as 33.32 N and from Gerber mean stress correction as 38.51 N, both at 100000 cycles. The Gerber mean stress correction results showed a very good agreement with the experimental result of 40.92 N, with around 6% error.

Comparing Figures 8 and 10 shows that, at high cycle fatigue, the stress cycle obtained from the elastic FE model provides an acceptable estimation of the fatigue limit of the plate at 100,000 cycles, largely due to failure driven by elastic strain. To reduce the model’s number of parameters (calibration of material parameters in the plastic domain) and computational costs, the developed elastic FE model can be directly used to investigate fatigue behavior of implants. Furthermore, comparing Figure 6 and Figure 7 reveals that the maximum stress location in the developed elastic FE model is similar to that of the experimental tests. This suggests that the selection of an elastic FE model to identify the maximum stress location was successful, which is consistent with the findings of previous studies (Babić et al., 2020; Parr et al., 2021; Shi et al., 2021), where fatigue failure location was also correctly estimated. For example, Babic et al. (2020) compared the results of their simulation to the experiments of Griza et al. (2013).

### 4.3 Constitutive analysis of cyclic stress behaviors

**FIGURE 11:**
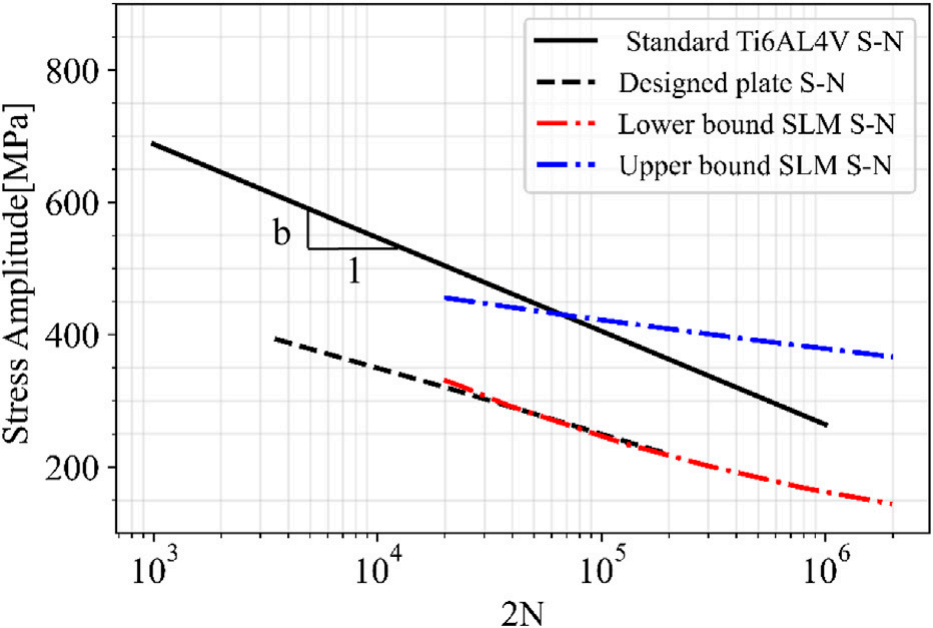
Stress *versus* number of cycles to failure for standard Ti6AL4V (when, $\sigma_{m}=0$ ), and designed plate compare to that of SLM Ti6AL4V specimens obtained by (Bezuidenhout et al., 2020), as-built (lower bound) and chemically polished surfaces (upper bound).

Furthermore, the minimum mechanical properties requirement of Ti6AI4V alloys produced by additive manufacturing according to ASTM F3001-14 were used in this study for the numerical methodology, along with a fabrication correcting factor *C*_*f*_ which impacts the generated S-N curve of the alloy to account for fabrication parameters. The results showed that the selected minimum mechanical properties in ASTM F3001-14 with a C_f_ = 1 led to a good prediction of fatigue life using Gerber’s method. However, the Goodman prediction led to an under-estimated fatigue life for the plate, confirming results of previous studies on classical fatigue theories using Goodman’s method (Sendeckyj, 2001; Asada et al., 2016; Liu et al., 2020). Figure 11 shows the stress *versus* number of cycles to failure for standard Ti6AL4V (when σ_m_ = 0) in black solid line. This line lies between the two curves obtained by Bezuidenhout et al. for SLM Ti6AL4V specimens with different post processing; as-built (lower bound) and chemically polished surfaces (upper bound) (Bezuidenhout et al., 2020). Furthermore, the comparison of the standard Ti6AL4V material and designed plate S-N curves in Figure 11 illustrates that the fatigue limit (at 10^6^) and the fatigue strength exponent *b* were both decreased for the plate S-N curve compared to the material curve. This difference is attributed to the stress concentration on sharp edges of the geometry, particularly at the joint between the plate bridge and screw holes, which is evidenced by the location of the maximum stress (see Figure 7) and the failure location of the plate experimentally (see Figure 6). Furthermore, the S-N curve of the material was obtained with zero mean stress (completely reversed stress cycle), whereas the plate is under stress cycles with mean stress. Therefore, the material S-N curve should be considered as an upper bound for the plate S-N curve.

The results obtained experimentally for the 3D printed Ti6Al4V plate are significantly influenced by the manufacturing process (SLM), the geometry of the plate, and loading conditions. The SLM process is known to produce parts with variable internal porosity and a rough surface (Benedetti et al., 2018). These characteristics introduce stress concentrations and notches which lead to a reduction in the fatigue life of the part (Leuders et al., 2013; Bordin et al., 2017; Almansoori et al., 2020). Additionally, the high-temperature flux induced by SLM during material melting can lead to residual stresses and anisotropy which can further reduce the fatigue limit (Edwards and Ramulu, 2014; Bartlett and Li, 2019).

The geometry of the part also impacts the fatigue limit, as sharp edges or material anisotropy introduced by SLM fabrication due to a specific part design, which can lead to stress concentrations. Furthermore, the loading conditions applied to the part can also influence the fatigue limit, as certain loading scenarios depending on their relative direction to the anisotropy direction, can result in crack propagation or higher stress concentrations, both of which reduce the fatigue life of the part (Almansoori et al., 2020; Jehn et al., 2020).

Previous attempts to use classical fatigue theories on medical implants have been made, as demonstrated by Ziaie et al. (2018), who estimated fatigue life for a dental implant without performing experiments and concluded that the results predicted by the Goodman method were in close agreement with clinical data. Senapl et al. (2001) also used ANSYS to evaluate the fatigue performance of five hip implant designs by determining a safety factor, but they did not validate their results through experiments. Therefore, in this study, to further validate the numerical estimation of fatigue life for the plates, experimental tests were conducted and the numerical results were found to be in good agreement with the experimental results. This study has demonstrated the potential of evaluating 3D-printed plates using Gerber’s method. However, further research is needed to assess the effects of stress concentration, manufacturing defects, and surface roughness on the fatigue performance of the plate. To this end, the S-N curve can be adjusted with a factor *C*_*f*_ to account for these factors and assess the accuracy of Gerber’s method under various geometric and fabrication conditions.

## 5 Conclusion

In this study, generic osteosynthesis plates were evaluated experimentally under in-service cyclic loading conditions, using a modified version of the ISO 12107 standard staircase method. It was found that performing tests on only 6 samples was sufficient to obtain accurate fatigue limit, compared with 12 samples with the standard staircase method. Moreover, the fatigue limit obtained from the numerical analysis was in agreement with the experimental results, indicating that the stress-life approach using Gerber mean stress correction and Basquin model is a reliable tool for estimating the in-service fatigue lifetime of such implants. This method provides significant advantages for optimizing patient-specific implant designs and reducing the need for expensive normative tests. Furthermore, the results showed that the minimum mechanical properties of 3D-printed Ti6AI4V alloy according to ASTM F3001-14 were sufficient for obtaining an accurate fatigue limit prediction. Additionally, it was determined that a linear elastic finite element model was sufficient for estimating the fatigue limit, while an elastic-plastic model provided a more accurate prediction throughout the implant’s cyclic life.

## Data Availability

The original contributions presented in the study are included in the article/supplementary material, further inquiries can be directed to the corresponding author.
